# Quantifying bluetongue vertical transmission in French cattle from surveillance data

**DOI:** 10.1186/s13567-019-0651-1

**Published:** 2019-05-14

**Authors:** Noémie Courtejoie, Laure Bournez, Gina Zanella, Benoît Durand

**Affiliations:** 1 Epidemiology Unit, Laboratory for Animal Health, ANSES (French Agency for Food, Environmental and Occupational Health and Safety), Paris-Est University, 14 Rue Pierre et Marie Curie, 94700 Maisons-Alfort, France; 2 Mathematical Modelling of Infectious Diseases Unit, Institut Pasteur, UMR2000, CNRS, 75015 Paris, France; 3 Nancy Laboratory for Rabies and Wildlife, ANSES (French Agency for Food, Environmental and Occupational Health and Safety), CS 40009, 54220 Malzéville, France

## Abstract

Bluetongue is a vector-borne disease of ruminants with economic consequences for the livestock industry. Bluetongue virus serotype 8 (BTV-8) caused a massive outbreak in Europe in 2006/2009 and re-emerged in France in 2015. Given the unprecedented epidemiological features of this serotype in cattle, the importance of secondary routes of transmission was reconsidered and transplacental transmission of BTV-8 was demonstrated in naturally and experimentally infected cattle. Here we used surveillance data from the on-going outbreak to quantify BTV-8 vertical transmission in French cattle. We used RT-PCR pre-export tests collected from June to December 2016 on the French territory and developed a catalytic model to disentangle vertical and vector-borne transmission. A series of in silico experiments validated the ability of our framework to quantify vertical transmission provided sufficient prevalence levels. By applying our model to an area selected accordingly, we estimated a probability of vertical transmission of 56% (55.8%, 95% credible interval 41.7–70.6) in unvaccinated heifers infected late in gestation. The influence of this high probability of vertical transmission on BTV-8 spread and persistence should be further investigated.

**Electronic supplementary material:**

The online version of this article (10.1186/s13567-019-0651-1) contains supplementary material, which is available to authorized users.

## Introduction

Bluetongue is a non-zoonotic vector-borne viral disease of domestic and wild ruminants notifiable under European legislation (Directive 2007/2075) and OIE rules [1]. Bluetongue virus (BTV) is a double-stranded RNA virus of the genus *Orbivirus* within the *Retroviridiae* family, with 27 known serotypes [2, 3]. Disease outcome varies depending on the serotype and species involved [4], ranging from the absence of clinical signs to death and abortion in the worst cases. BTV is mainly transmitted by biting midges of the genus *Culicoides*, but there is evidence for the direct transmission of at least some strains of BTV by transplacental, iatrogenic and oral transmission [5].

**Figure 1:**
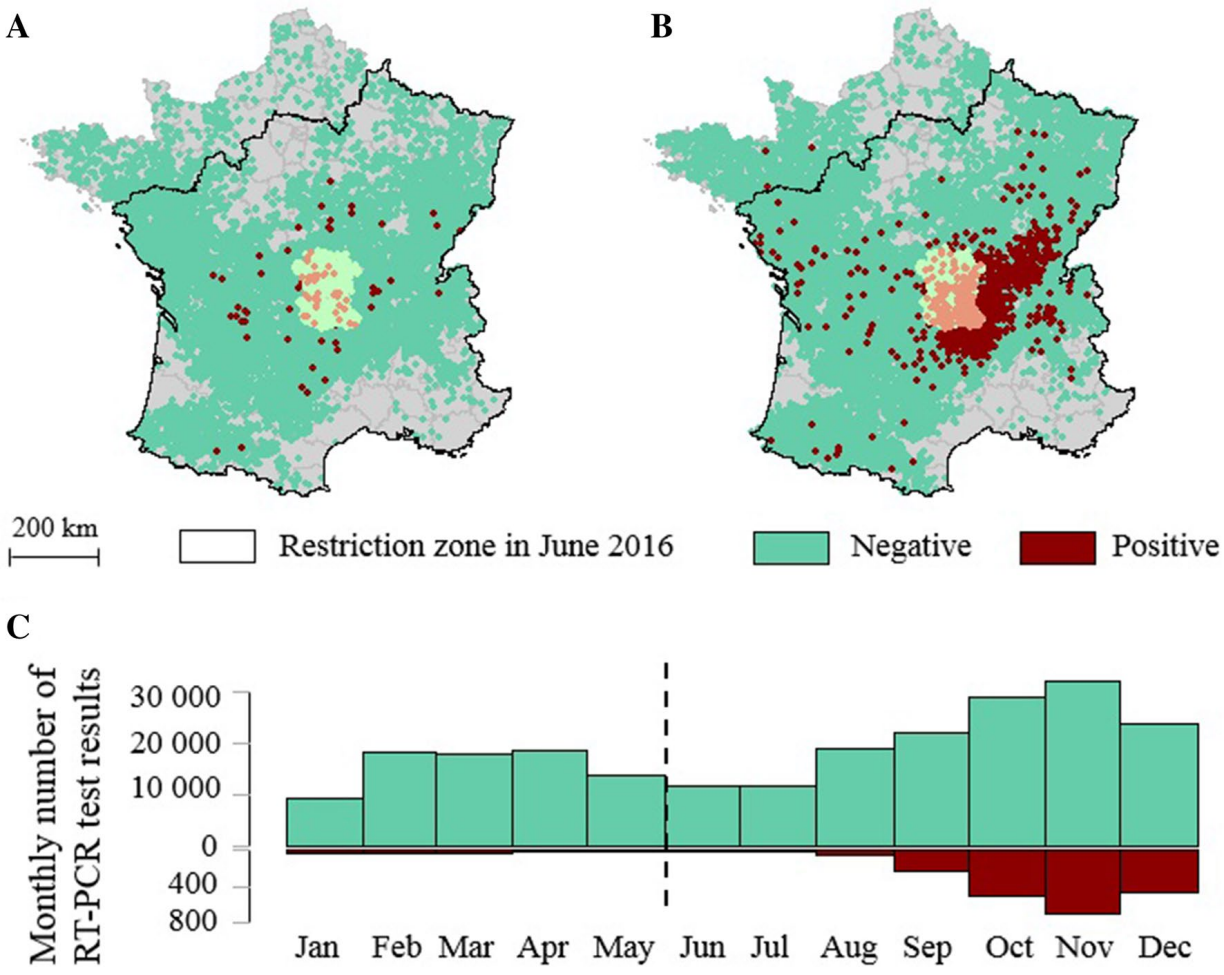
**Pre-export BTV RT-PCR results in cattle tested in France in 2016.** Pre-export BTV RT-PCR results: **A** from January 2016 to May 2016 (*N* = 78 074); **B** from June 2016 to December 2016 (*N* = 151 691). Positive and negative results are shown in lighter colors in *Allier* and *Puy*-*de*-*Dôme,* where BTV-8 substantially circulated in 2015. **C** Monthly distribution of RT-PCR test results in 2016, excluding *Allier* and *Puy*-*de*-*Dôme.*

In 2006, serotype 8 (BTV-8) was reported for the first time on the European continent, causing a massive outbreak throughout North-West Europe, eventually overcome in 2010 after several vaccine campaigns [6]. Although undetected in Europe for 5 years, BTV-8 reemerged in August 2015 in Central France, with a nearly identical viral strain to the one that had circulated in 2006/2009 [7]. BTV-8 has kept spreading since then with a clear increase in virus circulation between the 1^st^ and 2^nd^ year after the re-emergence [8]. The loss of the bluetongue free status consecutive to the 2015 BTV-8 re-emergence led to restricted conditions for cattle trade exchange in the restriction zone within 150 km of notified BTV-8 cases (Figure 1, Additional file 1).

The importance of secondary transmission routes in disease spread and persistence has been a subject of debate for years. During the 2006/2009 outbreak, an increase in the number of abortions in cattle was reported in the affected regions [9]. In addition, hydranencephalopathy was observed in aborted calves and lambs in Belgium and in the Netherlands in association with BTV-8 infection [10, 11]. These observations led to the hypothesis that the circulating BTV-8 strain differed from previous field strains in its ability to infect the fetus by crossing the ruminant placenta [10, 12].

A series of studies provided evidence of BTV-8 transplacental transmission in cattle, sheep and goats, from experimental infections [16, 17, 21–25, 26–28] and field observations of aborted fetuses, newborn and dam samples collected during the 2006/2009 European outbreaks [9–11, 13–15, 18–20]. In cattle, BTV-8 was even isolated in four vertically-infected newborns [13–15]. The proportion of viable calves born from infected dams that are infected through vertical transplacental transmission was quantified in experimental infections (< 10 observations, [23]), and in the analyses of up to a couple hundred of paired samples of dams and newborn calves (*N* < 110 [10, 15, 18, 20], 110 < N < 230 [14, 19]). This proportion, hereafter referred to as “vertical transmission”, was estimated to range from 16 to 42% [10, 14, 15, 18–20, 23] and the greatest values were obtained for infections occurring late in gestation [18, 20].

Vertical transmission has never been assessed by disentangling several modes of transmission from a large set of field observations. This can be achieved by adapting catalytic models such as those used to quantify the level and evolution in time of the force of infection of chikungunya [29], dengue [30] or bluetongue [31]. Vertical transmission in hosts is known to be possible in these diseases. Yet, only vector-borne transmission has been considered in the models developed so far [29–32].

Despite evidence of BTV vertical transmission, it is still considered as a secondary route of infection and has rarely been accounted for in the design of transmission models of disease spread [32]. Most of BTV spread throughout Europe is generally attributed to vector dispersal and the role of vertical transmission in BTV-8 spread and overwintering remains unknown.

Here we investigate whether vertical transmission can be estimated by disentangling several modes of transmission from a large set of field observations without using paired samples of dams and calves, but by inferring the dams’ probable infectious status. We used French surveillance data collected in 2016 for pre-export testing, performed by RT-PCR under various protocols (Additional file 1). Although vaccination was the main measure to secure animal movements, RT-PCR tests were additionally used after primo-vaccination to reduce the delay of animal movement to countries of the European Union. Calves (i.e. cattle < 12 months) were over-represented in this dataset given that they could not be vaccinated before 10 weeks of age, and given that Spain, the main importing country of French live calves, had signed an agreement to allow importation of cattle protected from *Culicoides* bites and tested negative by RT-PCR (Additional file 1). The high number of positive RT-PCR results in calves born from unvaccinated heifers during the 2016 season of virus circulation and sampled < 3 months of life (6.7% positive in *Saône*-*et*-*Loire*, *N* = 69 out of 1023 samples) suggested a cumulative exposure to both vertical and vector-borne transmission.

To further explore and quantify vertical transmission in viable newborn calves in this context, we first identified two populations within our dataset based on birthdates: we separated those that had only been exposed to BTV through vector bites from those that may have been additionally exposed to vertical transplacental transmission. Then, we built a catalytic model that allowed separating vertical and vector-borne transmission. We carried out a series of in silico experiments (i) to test that framework in a fully known population, under different assumptions of vertical transmission and different patterns of virus circulation; and (ii) to identify a set of conditions allowing reliable estimates to be obtained. We then selected an area meeting these conditions and applied our framework to real data to quantify vertical transmission in French cattle in 2016.

## Materials and methods

### Data and study area

The results of all RT-PCR pre-export tests carried out in 2016 on the French territory (Additional file 1) and registered in the database of the Ministry of Agriculture (SIGAL) were provided by the Ministry of Agriculture (*N* = 229 765) (Figure 1). RT-PCR tests allowing for the detection of all BTV serotypes were carried out by local laboratories and the positive ones were then specifically tested for BTV-8 [33]. The cut-off value of 40 threshold cycles (Ct) was used by all French laboratories. Doubtful results (positive in pan-BTV RT-PCR and negative in specific-BTV-8 RT-PCR or Ct-values > 35) were sent for confirmation to the National Reference Laboratory (ANSES, Maisons-Alfort).

All cattle sampled had been referenced in the National identification database (BDNI). We extracted their dams’ reference numbers. We screened the BDNI for the birthdates of all cattle and dams, for the herds visited by cattle up to their sampling date and for the herds visited by the dams up to their calving date. We did not know whether the dams had been infected or vaccinated against BTV-8. However, dams born after July 2011 were unlikely to have been vaccinated given that they were only exposed to the little implemented 2012 voluntary vaccination campaign [31, 34], following which vaccination was banned. We did not rule out the possibility of a low level BTV circulation between 2011 and 2015 given that: (i) anti-BTV antibodies had been detected as early as winter 2014 in calves born after the vaccination ban [35]; and (ii) low level circulation may have remained undetected due to an evolution of the circulating strain towards less-virulence [36]. However, we have shown in a previous study that potential BTV-8 circulation in cattle population in the area of the reemergence would have remained negligible until late 2015 [31]. Hence, we assumed that the dams born after July 2011 and that had not visited areas where bluetongue had mainly circulated in 2015 (i.e. *Allier* and *Puy*-*de*-*Dôme*) were likely to remain seronegative at least up to June 2016, and they were considered as “immunologically naive”.

### Data selection and cleansing

We aimed at identifying two populations within our dataset of cattle tested by RT-PCR, separating those that had only been exposed to BTV through vector bites during the 2016 season of virus circulation from those that may have been additionally exposed to vertical transplacental transmission during that season. The “season of BTV circulation” was included in the vector activity season, but limited to the time period when active and abundant *Culicoides* populations were considered able to spread BTV. The monitoring network for *Culicoides* populations implemented in France during fall and winter 2015/2016 indicated that vector activity progressively resumed across the territory from late March to mid-May 2016 [37]. However, BTV circulation only seems to have substantially resumed from summer onwards, as shown by the large increase in infectious herds detected from September alongside with an increase in viral loads in RT-PCR positive animals [8]. We thus assumed in the present study that the 2016 season of virus circulation did not substantially start earlier than June. We focused on that season and excluded all samples collected before June 1^st^ (*N* = 78 074, Figure 1A). The 96 positive samples (0.1%) among them probably indicated infection during the previous season of virus circulation, though they may also indicate low-level indoor vector activity of *Culicoides* during winter, one possible explanation for BTV overwintering [40].

**Figure 2:**
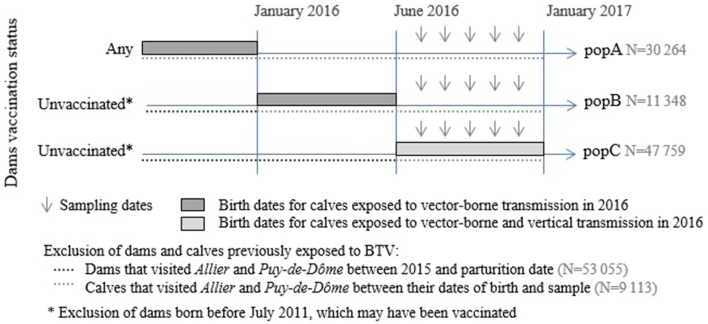
**Identification of cattle populations with contrasted exposure to vertical transmission.** In the dataset, three populations of cattle were identified based on birthdates and dams likely vaccinal status: cattle (popA) and calves (popB) that had only been exposed to BTV through vector bites vs calves (popC) that may have been additionally exposed to vertical transplacental transmission.

We then used the birthdates of the remaining cattle, the likely vaccination status of their dams and the list of farms where they have been kept to identify three sub-populations within the dataset (Figure 2). The two populations of cattle only exposed to vector bites (popA, popB) were made up of animals born before June 2016, considered to have no anti-BTV antibodies, neither colostral (maternal antibodies) nor self-acquired, at the beginning of the 2016 season of virus circulation: (i) to limit the possibility that animals would be protected by antibodies from the previous season, we excluded all cattle that had visited the areas in which BTV-8 had already substantially circulated in 2015, i.e. *Allier* and *Puy*-*de*-*Dôme* (*N* = 2539 in popA, *N* = 2032 in popB); (ii) we kept all those born before January 2016 (popA, Figure 2) as hypothetical colostral antibodies would most likely have disappeared by June 2016 [41]; and (iii) we kept those born between January and May 2016 (popB, Figure 2) only if they were unlikely to be protected by colostral antibodies, i.e. born from immunologically naive dams (*N* = 25 015 calves excluded from popB). The population of calves exposed to vertical transplacental transmission before their birth and to vector bites afterwards (popC, Figure 2) was made up of calves born after June 2016 from dams that may have been infected during gestation in the 2016 vector season: we thus kept only cattle born from dams considered as immunologically naïve at the beginning of the 2016 season of virus circulation (*N* = 28 040 calves excluded from popC). We excluded the calves of popC that had visited *Allier* or *Puy*-*de*-*Dôme* before being sampled (*N* = 4542). Eventually, we included 30 264, 11 348 and 47 759 samples for popA, popB and popC respectively (Figure 2).

### Estimation of vertical and vector-borne transmission

We developed a methodological framework to disentangle transplacental and vector-borne transmission. This framework aimed at estimating monthly probabilities of vector-borne infection from June to December 2016, and a unique probability of vertical transmission considered constant over the 7 months of the study period. We extended existing catalytic models built for the analysis of age-stratified serological data [29–31] to the analysis of age-stratified RT-PCR data, assuming that animals infected by either vertical or vector-borne transmission within the study period would stay RT-PCR positive for 4 months (starting from the date of infection in the dam in case of vertical transmission). For simplification, the RT-PCR tests were considered perfect for BTV-8 detection [42].

We denoted $\lambda \left( t \right)$ the force of vector-borne infection at time *t*, i.e. the instantaneous risk for a susceptible animal to get infected through vector bites at time *t*. All animals were considered as directly exposed to the time-varying forces of vector-borne infection: (i) from June 2016 or from their birthdate (whichever was later) to their sampling date for cattle, and (ii) from June 2016 to their calving date for the dams of calves born after June 2016 (popC, Figure 2). Calves from popC (Figure 2) were additionally exposed to vertical transmission before their birthdate, with γ the constant probability of BTV transmission from dams infected during gestation to their progeny.

**Figure 3:**
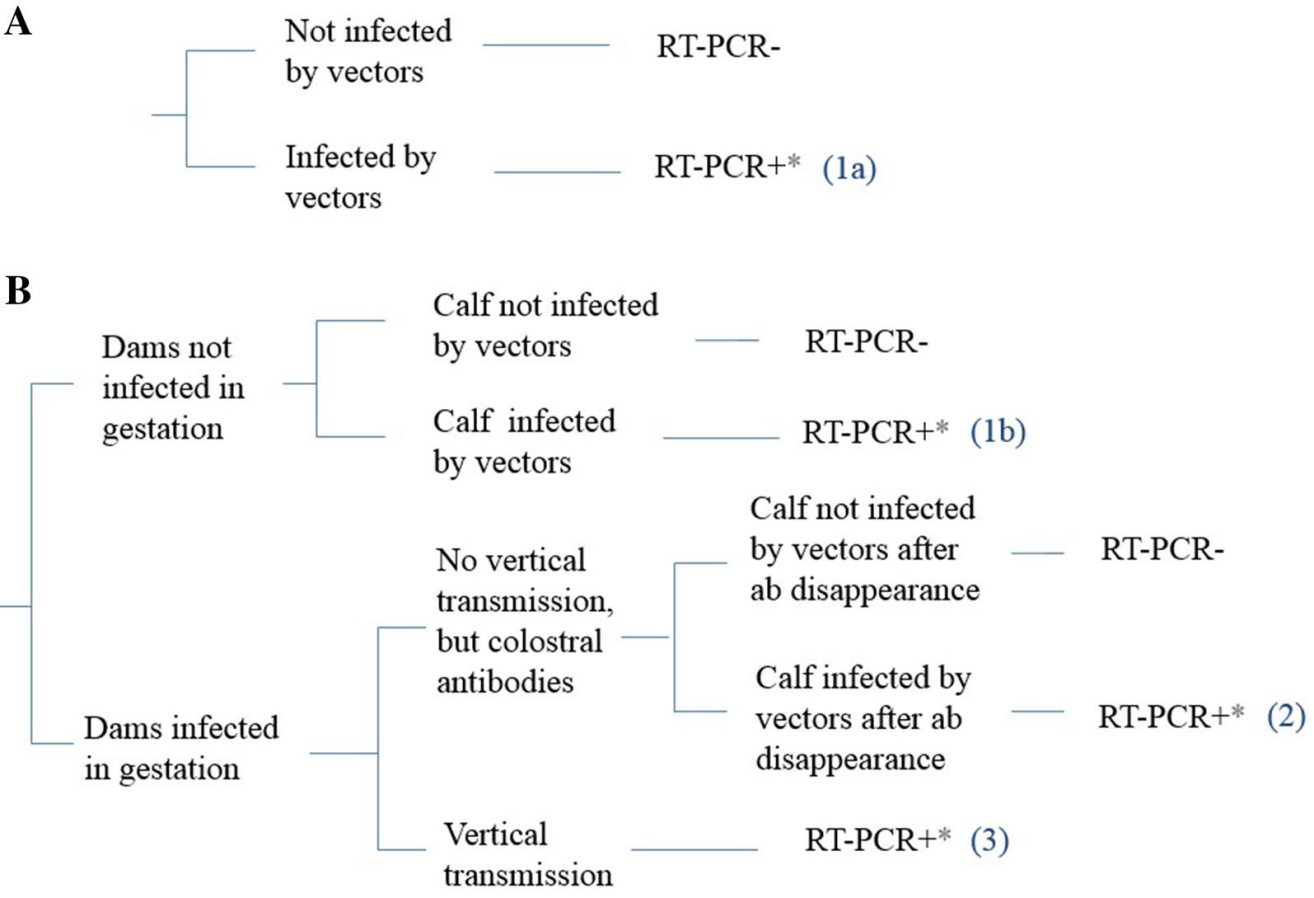
**Infection pathways in cattle differentially exposed to vector bites and vertical transmission.** Infection parthway: **A** for calves only exposed to vector-borne transmission (popA and popB), **B** for calves exposed to both vector-borne and vertical transmission (popC). ab, antibodies; *, within the length of RNA detection in blood.

We identified three possible infection pathways (Figure 3): (*1a*, *1b*) vector-borne, in cattle exposed to infectious bites from June 2016 (or their birthdate) to their sampling date; (2) vector-borne, in calves born from dams infected during gestation that transmitted colostral antibodies but not BTV and exposed to infectious bites from the disappearance of colostral antibodies to their sampling date; (3) transplacental, in calves born from dams infected during gestation and exposed from June 2016 to calving. Pathway *1a* applied to all cattle only exposed to vectors (popA, popB, Figure 2), and pathway *1b* applied to those additionally exposed to vertical transmission, provided their dams had not been infected during gestation (popC, Figure 2).

We expressed the likelihood function as the product of the likelihoods for each population:

$$L = L_{A} *L_{B} *L_{C}$$

where *L*_*A*_, *L*_*B*_ and *L*_*C*_ are the likelihoods for popA, popB and popC (Figure 2), respectively.

The likelihoods for popA and popB are the same, that is:

$$L_{A,B} = \prod\limits_{i \in Pos} {P\left( {vector} \right)*\prod\limits_{i \in Neg} {1 - P\left( {vector} \right)} }$$

where *Pos* and *Neg* denote animals with positive or negative RT-PCR results when sampled.

The probability of animal *i* from popA or popB of being infected by vectors within the length of detection of BTV RNA in blood (i.e. infection through pathway *1a*) is:

$$P\left( {vector} \right) = \int\nolimits_{{max(t_{0} ,\:t_{S}^{( i)} - t_{D} ) }}^{{t_{S}^{( i)} }} {\lambda \left( \tau \right)\exp \left( { - \mathop \int \nolimits_{{t_{0} }}^{\tau } \lambda \left( t \right)dt} \right)d\tau }$$

where *t*_*S*_ is the time of sampling; *λ*(*t*), the force of infection at time *t*; *t*_0_, the start of the study period (i.e. June 1^st^ 2016); and *t*_*D*_, the time period over which an infected animal is RT-PCR-positive.

The likelihood for popC, where calves could be infected either by vectors or by vertical transmission, is:

$$\begin{aligned} L_{C} &= \prod\limits_{i \in Pos} [ P({vector,dam\,not\,infected} ) + P ( {vector,dam\,infected} ) + P ( {vertical}) ] \\ & \quad * \prod \limits_{i \in Neg} 1 - [ P ( {vector,dam \,not\, infected} ) + P ( {vector,dam\,infected} ) + P( vertical ) ]. \end{aligned}$$

The probability of animal *i* being born to an uninfected dam, and infected by vectors within the length of RNA detection (i.e. infection through pathway *1b*) is:

$$P\left( {vector, dam\,\,not\,\,infected} \right) = exp\left( { - \int\nolimits_{{t_{0} }}^{{t_{B}^{( i)} }} \lambda \left( t \right)dt} \right)* \int\nolimits_{{\text{max} \left( {t_{B}^{( i )} , \:t_{S}^{( i)} - t_{D} } \right)}}^{{t_{S}^{( i)} }} \lambda \left( \tau \right)\exp \left( { - \int\nolimits_{{t_{B}^{( i)} }}^{\tau } \lambda \left( t \right)dt} \right)d\tau$$

where *t*_*B*_ is the time of birth.

The probability of animal *i* being born to an infected dam without being infected vertically, and infected by vectors within the length of RNA detection (i.e. infection through pathway *2*) is:

$$P\left( {vector, dam\,\, infected} \right) = \left( {1 - \gamma } \right)\left[ {\int \nolimits_{{t_{0} }}^{{t_{B}^{( i)} }} \lambda \left( \tau \right)\exp \left( { - \int \nolimits_{{t_{0} }}^{\tau } \lambda \left( t \right)dt} \right)d\tau } \right]*\left[ {\int \nolimits_{{\text{max} \left( {t_{B}^{( i)} + t_{C} , \:t_{S}^{( i)} - t_{D} } \right)}}^{{t_{S}^{( i)} }} \lambda \left( \tau \right)\exp \left( { - \int \nolimits_{{t_{B}^{( i)} + t_{C} }}^{\tau } \lambda \left( t \right)dt} \right)d\tau } \right]$$

where *t*_*C*_ is the duration of colostral antibodies.

Finally, the probability of animal *i* being infected by vertical transmission and sampled within the length of RNA detection (i.e. infection through pathway *3*) is:

$$P\left( {vertical} \right) = \left\{ \begin{array}{ll} \gamma \int \nolimits_{{\text{max} \left( {t_{0} , \:t_{S}^{( i)} - t_{D} } \right)}}^{{t_{B}^{( i)} }} \lambda \left( \tau \right)\exp \left( { -\int \nolimits_{{t_{0} }}^{\tau } \lambda \left( t \right)dt} \right)d\tau & \quad\left( {t_{S}^{( i )} - t_{D} \le t_{B}^{( i )} } \right) \\ 0 & \quad (t_{S}^{(i)} - t_{D} > t_{B}^{( i )} ) \\ \end{array} \right.$$

We assumed a duration of 4 months for *t*_*D*_ and *t*_*C*_, and varied these values in a sensitivity analysis. In practice, we modelled the force of vector-borne infection with a step function equal to $\lambda_{j}$ during month *j *= 1,…*N,* with *N*, the total number of months in the study period.

We estimated all parameters by fitting the model in a Bayesian Markov chain Monte Carlo (MCMC) framework with the No-U-Turn Sampler [43] implemented in R (RStan package version 2.14.2 [44]). Parameters *λ*_*j*_ had a lognormal prior distribution (μ = − 2, σ^2^ = 5) to explore preferentially small values, and parameter *γ* had a non-informative beta prior distribution (α = 1, β = 1). We used the following settings: 2 chains, 3000 iterations per chain, warmup of 1500, thin of 1.

### In-silico experiments

We tested our framework with a series of toy examples presented in Additional file 2. In summary, we constructed fully known populations in which we varied the values of four key parameters in order to investigate their influence on the estimation of vertical and vector-borne transmission: the probability of vertical transmission in the population, the size of the dataset, the level of exposure to vector-borne transmission, and the spatial heterogeneity of that exposure in the study area.

### Application to real data

#### Selection of the study area

The in silico experiments (Additional file 2) allowed identifying a set of conditions providing reliable estimates of vertical transmission. The key criterion was the global level of exposure to infectious bites over the study period, approximated by the RT-PCR proportion of positive results in cattle only exposed to vector-borne transmission, calculated from June to December 2016 (RT-PCR + %). In our simulations, we obtained the best results when the proportion of RT-PCR positive samples was above 10%, a threshold that applies to our sampled population as the in silico experiments were conducted in a synthetic population with similar age-class proportions and sampling dates. Over that threshold, and for the values tested, no other parameter impacted the mean estimates of vertical transmission. Smaller data sizes (while ≥ 2500) resulted in unbiased estimates but with an increase of credible intervals.

In our dataset, we thus looked for an area with a high level of vector-borne transmission, approximated by RT-PCR + % in cattle only exposed to vector bites (popA and popB, Figure 2). In a first step, we excluded samples from areas with little or no BTV circulation in 2016. This was achieved by dividing the French territory in 20 km × 20 km grid cells, and applying the following exclusion criteria: (i) cells with ≤ 5% RT-PCR positive results; (ii) cells with ≤ 1 positive RT-PCR sample. We then split this area in two, based on geographical criteria as circulation may have differed in closer proximity to *Allier* and *Puy*-*de*-*Dôme*, where BTV-8 had substantially circulated in 2015. We checked whether the pre-identified study conditions were met in the whole area as well as in both sub-areas.

#### Estimation of vector-borne and vertical transmission in the selected area

We investigated vector-borne and vertical transmission using all cattle from the selected area. We compared inference results obtained with or without accounting for spatial heterogeneity in exposure, using respectively: (i) model one-area, allowing the reconstruction of a unique set of monthly probabilities of vector-borne infection in the whole area and a unique probability of vertical transmission; and (ii) model sub-areas, allowing the reconstruction of distinct sets of monthly probabilities of vector-borne infection for each sub-area, but a unique probability of vertical transmission for both sub-areas. As a control, we also analyzed the two sub-areas separately, providing area-specific probabilities of vector-borne infection and vertical transmission. We used these results to estimate the numbers of calves in the dataset that had been infected during gestation.

#### Sensitivity analysis

We conducted a sensitivity analysis to evaluate the impact on inference results of modelling assumptions regarding the length of BTV RNA detection in blood and the length of persistence of colostral antibodies. We used *model one*-*area.* Both lengths had been set at 4 months in the model and we tested the additional values of 2, 3, 5 and 6 months for both of them.

## Results

### Selection of an area with a high level of exposure to infectious bites

**Figure 4:**
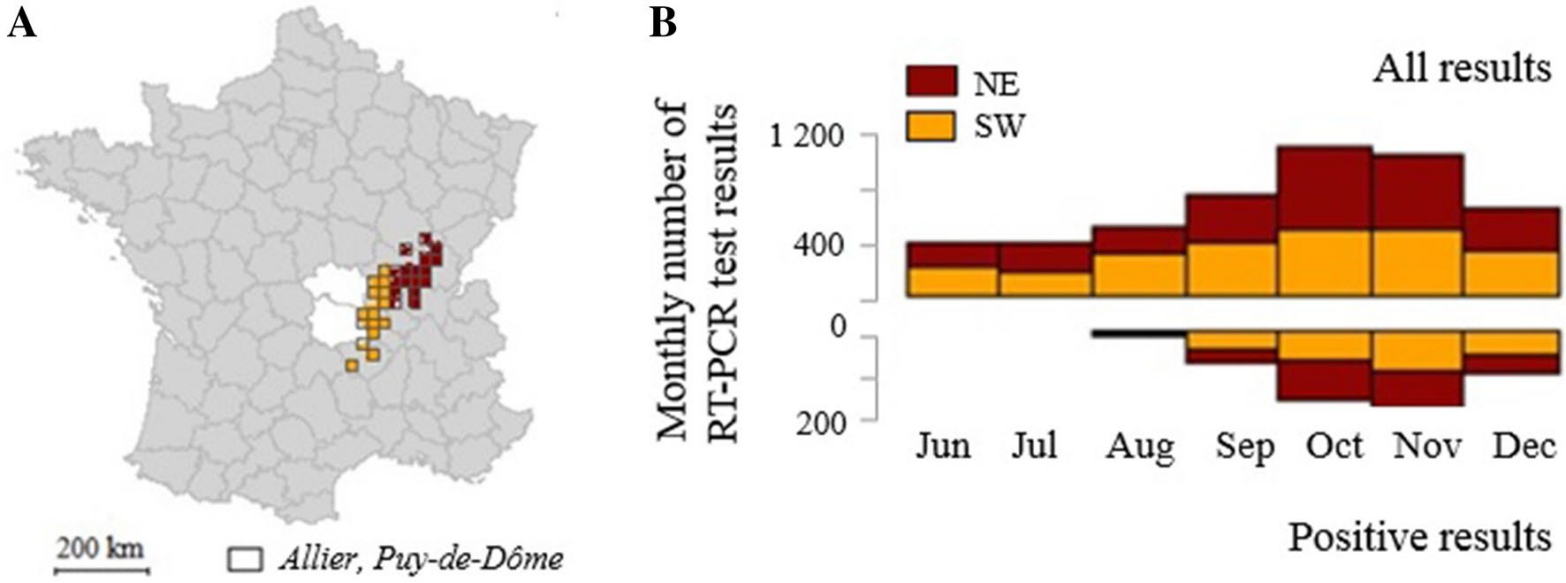
**Study area with high level of vector-borne virus circulation from June to December 2016, France. A** Map of RT-PCR results in cattle meeting the inclusion criteria (*N* = 5061), broken down in two sub-areas based on proximity to *Allier* and *Puy*-*de*-*Dôme*: the NE and SW areas; **B** histograms of the monthly number of RT-PCR samples in the NE and SW areas from June to December 2016. NE, North–East; SW, South–West.

The in silico experiments stressed the need to select an area with a high level of vector-borne virus circulation, without reducing too strongly dataset size. We selected a study area by applying the exclusion criteria defined above (section “Selection of the study area”), thus removing parts of the French territory with little or no RT-PCR + results. In the remaining area (Figure 4A), we had 2216 samples from cattle only exposed to vector-borne transmission and the RT-PCR + % reached 15.6% (> 10%). The overall dataset size, considering the whole set of cattle meeting the inclusion criteria defined above (section “Data selection and cleansing”) was 5061 (> 2500) (Figure 4B, Table 1). We additionally subdivided this area into two geographical sub-areas, referred to as the North–East (NE) and South–West (SW) areas, the latest being closer to *Allier* and *Puy*-*de*-*Dôme* (Figure 4A). Both areas showed high RT-PCR + % (> 10%); slightly more in the NE than in the SW area, though not significantly (*p* = 0.31, χ^2^ test) (Table 1). The overall dataset sizes were close to the smallest value tested in the in silico experiments: *N* = 2468 in NE and *N* = 2593 in SW (Table 1).

**Table 1:** **Proportion of RT-PCR positive results in the study population calculated from June to December 2016 **

Study area	Total n° cattle	N° cattle only exposed to vectors	RT-PCR + % in all sampled cattle (N° RT-PCR +)	RT-PCR + % in sampled cattle only exposed to vectors (N° RT-PCR +)
NE	2468	1255	12.7% (314)	16.3% (205)
SW	2593	961	11.0% (285)	14.7% (141)
Total	5061	2216	11.8% (599)	15.6% (346)

The proportions of RT-PCR positive cattle were calculated in all sampled cattle and in sampled cattle only exposed to vector-borne transmission (popA and popB), in the whole study area and in the NE and SW areas separately.

NE: North–East; SW: South–West; N°: number of; RT-PCR + %: proportion of positive results by RT-PCR, calculated over the whole period from June to December 2016.

### Inference results in real data

**Figure 5:**
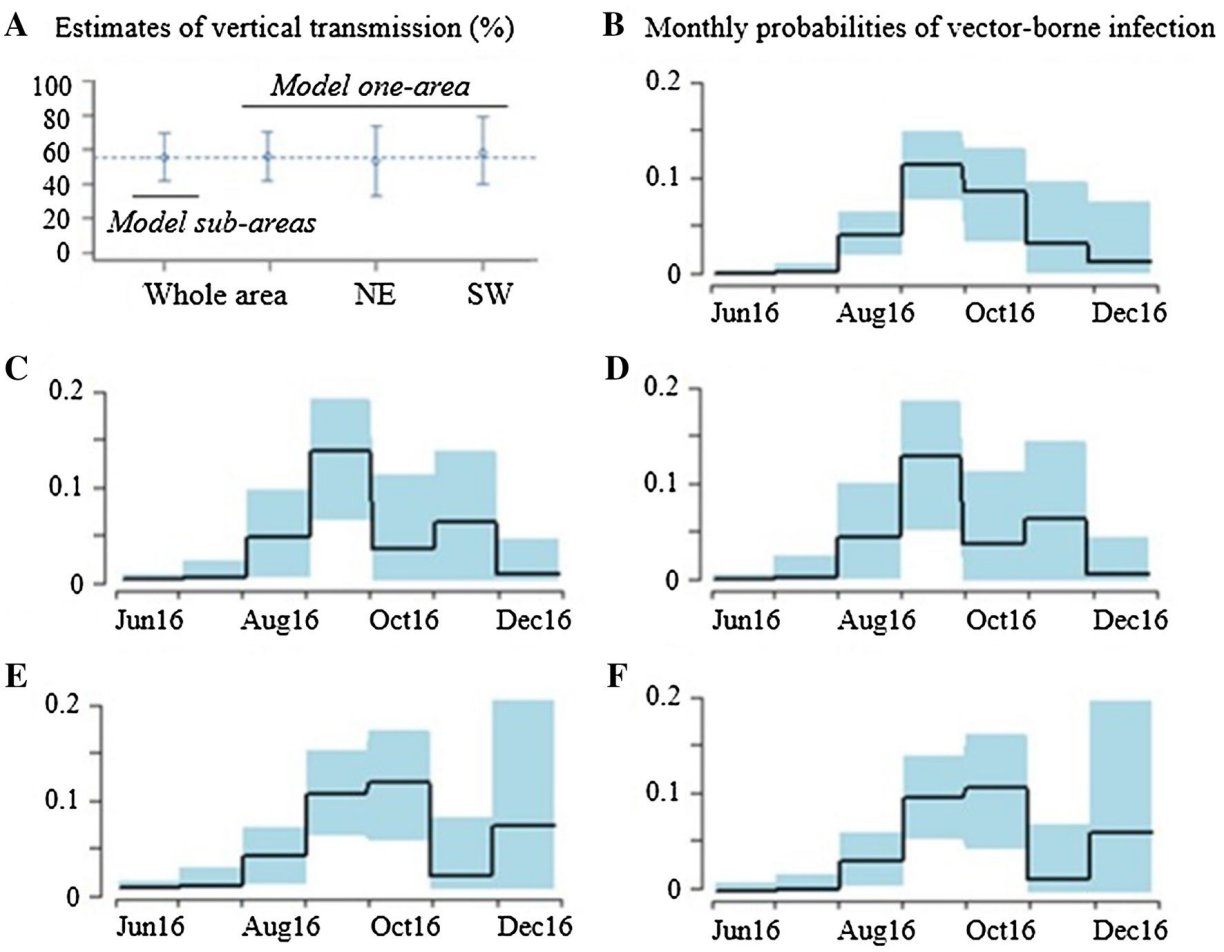
**Inference results: estimates of vertical and vector-borne transmission. A** Estimation of vertical transmission with models *one*-*area* or *sub*-*areas*, in the whole area or in areas NE and SW separately. **B**–**F** Reconstruction of the monthly probabilities of infection via vector-borne transmission (and their CI 95%), in the whole area (**B**), in NE (**C**, **D**) and SW (**E**, **F**) separately with model *sub*-*areas* (**C**, **E**) and *one*-*area* (**D**, **F**). NE, North–East, SW, South–West.

The MCMC chains correctly converged (Additional file 3). In the whole study area, we estimated similar probabilities of vertical transmission with models *one*-*area* and *sub*-*areas*: 55.8% (CI 95% 41.7–70.6) and 55.2% (CI 95% 41.7–69.6) respectively (Figure 5A). *Model one*-*area* only allowed reconstructing the overall probabilities of vector-borne infection (Figure 5B) while *model sub*-*areas* provided area-specific patterns (Figures 5C and E).

Our estimates of vertical transmission were validated by running *model one*-*area* in each area separately, thus providing two independent estimates in the NE and SW areas, that were similar to those estimated in the whole area but with wider credible intervals: 53.3% (CI 95% 33.0%–73.6%) in the NE area, and 57.8% (39.5%, 78.8%) in the SW area (Figure 5A). We also obtained similar area-specific patterns of probabilities of vector-borne infection (Figures 5C–F for the NE and SW areas respectively).

We applied the reconstructed probabilities of vector-borne infection to gestating heifers from the study area that gave birth after June 2016 (*N* = 2845). We estimated that 296 (CI 95% 256–339) of them were likely to be RT-PCR positive at their calving date, resulting in 163 (CI 95% 141–187) vertically infected calves, i.e. 27.2% of all RT-PCR positive cattle and 64.4% of RT-PCR positive calves born during the 2016 season of virus circulation (Table 2). These proportions seemed slightly higher in the SW vs NE area, but the difference was not significant (*p* = 0.12, χ^2^ test).

**Table 2:** **Estimation of the number (and proportion) of calves infected by vertical transmission in the study area**

Study area	N° predicted RT-PCR + dams [CI 95%]	N° predicted vertically infected calves [CI 95%]	% of RT-PCR + attributable to vertical infection [CI 95%]	
			In all cattle	In cattle exposed to vertical transmission
NE	139 [113–167]	77 [62–92]	24.5% [19.7–29.3]	70.6% [56.9–84.4]
SW	157 [128–189]	87 [71–104]	30.5% [24.9–36.5]	60.4% [49.3–72.2]
Total	296 [256–339]	163 [141–187]	27.2% [23.5–31.2]	64.4% [55.7–73.9]

NE: North–East; SW: South–West; N°: number of.

**Figure 6:**
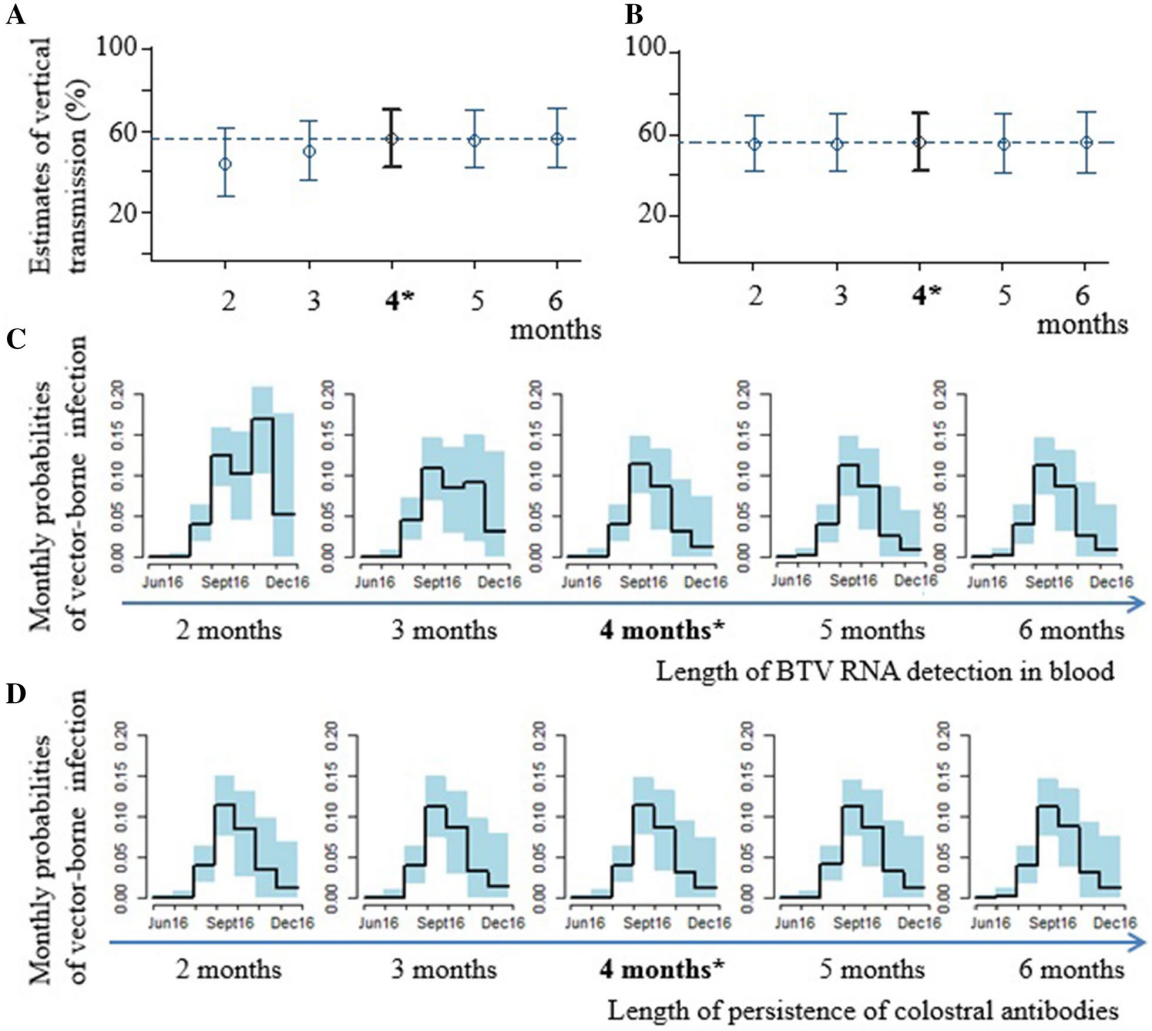
**Sensitivity analysis for two lengths: BTV RNA detection in blood and persistence of colostral antibodies.** Impact on estimates of vertical transmission (mean value and CI 95%) of the length of BTV RNA detection in blood (**A**), and of the length of persistence of colostral antibodies (**B**); Impact on estimates of monthly probabilities of vector-borne infection (and CI 95%) of length of BTV RNA detection in blood (**C**) and of length of persistence of colostral antibodies (**D**). The estimates of vertical transmission and probabilities of vector-borne infection were obtained with the same model (*one-area*), but with variations in both lengths considered. The base-line values used for these lengths in all other analyses are the ones in bold (*). The other values are those investigated as part of the sensitivity analysis. *, Base value used in all other analyses in the present study.

The sensitivity analysis conducted with *model one*-*area* showed no impact of the lengths of persistence of colostral antibodies on inference results (Figures 6B and D). However, for lengths of BTV RNA detection in blood less than 4 months, we obtained slightly lower estimates of vertical transmission (Figure 6A): 49.8% (CI 95% 35.7–65.0) for 3 months, and 43.5% (CI 95% 27.6–61.4) for 2 months with an additional impact on the reconstructed probabilities of vector-borne infection (Figure 6C).

## Discussion

In this paper, we quantified bluetongue vertical transmission in French cattle from surveillance data. We used RT-PCR pre-export tests collected from June to December 2016 on the French territory and developed a catalytic model to disentangle vertical and vector-borne transmission. Vertical transmission had already been quantified in specific settings with few observations such as experimental infections or analyses of paired samples of calves and dams, but never from nation-wide surveillance data.

Our modelling framework was first tested in in silico experiments carried out in synthetic populations with similar distributions of age-classes and sampling dates to that of the sampled population: (i) we showed its ability to reconstruct the probability of vertical transmission and the monthly probabilities of vector-borne infection; and (ii) we identified a set of study conditions providing reliable estimates. The level of exposure to infectious bites was identified as the most influential parameter, with the poorest estimates of vertical transmission obtained in the lowest exposure scenarios. The proxy that we used for the level of exposure to infectious bites also depended on the timing of infection, which we did not vary in these experiments as we expected most transmission to occur between September and October in all areas with substantial BTV-8 circulation in 2016 [8]. For a given level of exposure, we also highlighted an impact of dataset size as we obtained wider credible intervals with smaller datasets. However, the model performed equally with the three probabilities of vertical transmission tested, and inference did not seem sensitive to heterogeneity in the levels of vector-borne virus circulation in the study area; though the model considering area-specific patterns provided additional information on spatio-temporal contrasts of vector-borne virus circulation.

Accordingly, we selected a study area that we split into two geographical sub-areas in which we reconstructed slightly different levels of vector-borne virus circulation (Figure 5). Overall, we estimated 56% vertical transmission from infected gestating heifers to their calves, so that 64% of the RT-PCR positive calves born after June 2016 had likely been vertically infected. The robustness of the inference results was confirmed by obtaining similar estimates in the whole area and in sub-areas, and with both models accounting or not for heterogeneity in exposure to infectious bites.

The ability of the BTV-8 strain that circulated in Europe from 2006 to 2010 to cross the placental barrier in cattle had already been proven in various studies. Transmission from dams infected during gestation to their calves was quantified, varying from 16 to 42% [10, 14, 15, 18–20, 23]. However, these proportions were estimated in a variety of settings: experimental infection [23] or field observations using calves and dams paired samples [10, 14, 15, 18–20]; they were based on RT-PCR [14, 15, 18–20, 23] and/or ELISA tests [10, 14, 19]; performed on fetuses extracted 3 weeks after experimental infection [23], pre-colostral sera sampled during cesarean delivery [10, 14], or from calves sampled within the 1^st^ weeks [15, 18] or months [19, 20] of life. Moreover the timing of infection within gestation differed in these studies and there is evidence that infection in the early stages of fetal development may result in abortion [11, 14] and/or severe brain defects such as hydranencephalies [10, 11]; whereas fetal infection later in gestation could more likely result in the birth of RT-PCR positive calves [19].

In the studies where vertical transmission was quantified, estimates were computed from newborn calves, except in van der Sluijs et al. [23] where dams were euthanized and fetuses extracted, showing 20% of transmission (CI 95% 3–56) for an infection in the first half of gestation. In larger studies based on field observations and in which dams could have been infected at various dates within gestation, similar and higher proportions were found: 16% (CI 95% 11–21, [20]), 21% (CI 95% 9–32, [19]), 33% (CI 95% 22–47 [15]), and 37% (CI 95% 28–47 [10]). Of interest, the probability of transplacental transmission was shown to increase significantly with the stage of gestation during which the dam became infected [15], and cattle that seroconverted in the second half of gestation had a 15.5 times higher chance of delivering an RT-PCR-positive calf compared to those that seroconverted in the first half of gestation [20]. This could explain the high estimates of 42% vertical transmission (CI 95% 22–63) found in dams that had been infected within the 2^nd^ and 3^rd^ trimester [18]; and 36% (CI 95% 18–57) when keeping only the results from dams infected in the second half of gestation in Santman-Berends et al. [20].

Estimates of vertical transmission thus varied given the study population and epidemiological context. In our setting: (i) we had information on dams that gave birth to viable calves, but nothing on abortions or stillbirths; (ii) all heifers were considered seronegative at the beginning of the study period; and (iii) all infected heifers had most likely been infected within the last 4 months of gestation given that we showed little vector-borne virus circulation from June to August 2016 in the study area (Figures 2 and 5). Our estimation thus applies to infection occurring late in gestation in heifers that had not been previously vaccinated nor infected, and allowing the birth of viable calves. The probability of 56% vertical transmission that we estimated is higher but consistent with the existing literature given our specific study context. We showed that our estimate would be less for shorter lengths of BTV RNA detection in blood. In addition, this probability may be less in the whole cattle population where some heifers could have been vaccinated, which is expected to reduce the risk of transplacental transmission [23]. It is also of note that the strain from the 2015 re-emergence has resulted in very little clinical infection so far, which may be explained by pathogen evolution towards a less virulent strain, as shown in an experimental infection in sheep with the 2015 vs 2007 BTV-8 strains [36]. The high probability of vertical transmission estimated from healthy calves may thus be explained by a limited ability of the virus to cause severe pathology, possibly resulting in more viable infected fetuses.

The catalytic model developed here has also proven useful in identifying the periods of virus circulation due to vector-borne transmission in cattle from the study area in 2016. This could not be estimated from monthly RT-PCR proportions of positive results alone as viral RNA can still be detected a few months after infection [38]. We identified that the virus mainly circulated in September and October, though the estimated pattern of virus circulation depended on the length of BTV RNA detection in blood. We highlighted contrasts in the monthly probabilities of vector-borne infection in the two sub-areas, with no significant differences. Larger datasets may have been needed to reduce the width of credible intervals.

We used catalytic models originally developed to analyze serological data. They rely on the fact that once an individual becomes positive, it remains so until the end of its follow-up period; and time represents its cumulative exposure. While antibodies against BTV persist in the long-term, BTV RNA can only be detected during a smaller time window. In our model, we thus added a 4 months period, calculated from the date of vector-borne infection in the calves or their dams, after which we assumed that RT-PCR test results would be negative. We showed in a sensitivity analysis that lengths of BTV RNA detection in blood of 3 months and less would impact inference results. However, BTV RNA has been shown to be detected by RT-PCR in blood cells of cattle for up to 6 months after infection [38, 39]. As this period may differ in newborns, follow-up studies were conducted on calves that were RT-PCR positive at birth [15, 19]. The ten calves followed-up by Darpel et al. [15] became negative on average 3.1 months (CI 95% 1.7–5.2) after their birth; and Santman-Brends et al. [19] showed that calves could remain RT-PCR positive up to 5 months after birth. They even suggested that fetal infection late in gestation could result in the birth of RT-PCR positive calves up to 6 months after infection of the dam, and that the RT-PCR-signal remained longer in the newborn calves than in the dams themselves. Hence, the 4 months length of BTV RNA detection that we used for modelling is consistent with median values found in the literature, though we did not account for inter-individual variability.

Here we provided estimates of vertical transmission in French cattle in 2016 for infections occurring late in gestation and allowing the birth of viable calves. The high probability of 56% highlights that this transmission route may be more widespread than expected, though its true epidemiological impact remains to be assessed. There is still no evidence that vertically infected calves can further transmit BTV-8 to *Culicoides* vectors, nor that they can keep long-term antibodies. More focus should be given to this transmission route to help understanding BTV-8 spread, overwintering and reemergence in France after 5 years without being detected.

## Additional files

**Additional file 1.** **Description of cattle trade protocols in the restriction zone.** This paragraph gives more details about the control measures that were implemented during the study period (2016), and provides a better understanding of the data collection process.

**Additional file 2.** **In-silico experiments to test the modelling framework in fully known populations.** This file contains the protocol and results of the in silico experiments conducted to test our modelling framework. We constructed fully known populations in which we varied the values of four key parameters in order to investigate their influence on the estimation of vertical and vector-borne transmission: the probability of vertical transmission in the population, the size of the dataset, the level of exposure to vector-borne transmission, and the spatial heterogeneity of that exposure in the study area. We thus identified a set of conditions providing reliable estimates of vertical transmission.

**Additional file 3.** **Convergence of the chains obtained for the seven estimated parameters of model** ***one*-*area***. In this file, we checked the convergence of the chains obtained for the seven parameters. We first provided visual indicators of the convergence of the chains obtained for the seven parameters of model *one*-*area* in the whole study area. Then, we provided two convergence statistics for these chains: Rhat, that is the potential scale reduction factor and Neff, that is the effective number of samples.

## Data Availability

The datasets analyzed in the current study were generated by surveillance programmes initiated by the Ministry of Agriculture and are therefore not publicly available. They are, however, available from the Ministry of Agriculture upon request.

## Abbreviations

BTV: bluetongue virus
Ct: cycle threshold
CI 95%: 95% credible intervals
NE: North–East
RNA: ribonucleic acid
RT-PCR: reverse transcription polymerase chain reaction
SW: South–West
