# Supplementary material for: Quantifying bluetongue vertical transmission in French cattle from surveillance data
Source: Vet Res. 2019 May 14;50:34. doi: 10.1186/s13567-019-0651-1 (PMC6518818; doi:10.1186/s13567-019-0651-1)
Supplement: Supplementary file 1 — Additional file 1. Description of cattle trade protocols in the restriction zone. This paragraph gives more details about the control measures that were implemented during the study period (2016), and provides a better understanding of the data collection process. [file 13567_2019_651_MOESM1_ESM.docx]

**Additional file 1:** Description of cattle trade protocols in the restriction zone.

France is the first European exporting country of live cattle with over one million of head exported annually. In 2016, this included 1.1 million of live cattle >80 kg mainly exported for fattening to Italy (820 000) and Spain (145 000), and 169 000 calves <80 kg, 95% of which were exported to Spain (France Agrimer). In 2016, animals from the restriction zone (Figure 1) were subject to restricted conditions for cattle trade exchange. During the period of vector activity, vaccination against BTV-8 was the main measure to secure animal movements. Derogation protocols existed, relying on pre-export RT-PCR tests. A negative RT-PCR test was required to export vaccinated cattle from the restriction zone to countries of the European Union or to areas of France still free of BTV-8 before the required delay of 60 days following primo-vaccination (CE 1266/2007). Specific protocols were signed with Italy, Spain and Luxembourg to further reduce this delay given the vaccine type; from September 2016, it became ten days for all vaccines (DGAL/SDSPA/2016-281, 765, 504). As the presence of colostral antibodies may impair vaccination efficiency (Vitour et al., 2011), vaccination was banned for calves <2.5 months, meaning that with the additional delay after primo-vaccination, calves would not be able to leave the restriction zone before being 5.5 months old. A specific protocol was therefore established with Spain to allow the importation of French cattle protected against *Culicoides* bites during 14 days before leaving the restriction zone and tested negative by RT-PCR (DGAL/SDSPA/2016-281). For intra-national movements, animals were also allowed to leave the restriction zone if there were protected against *Culicoides* bites and tested negative by RT-PCR before and after leaving their herd (DGAL/SDSPA/2016-281, 765, 504).
