# Supplementary material for: Quantifying bluetongue vertical transmission in French cattle from surveillance data
Source: Vet Res. 2019 May 14;50:34. doi: 10.1186/s13567-019-0651-1 (PMC6518818; doi:10.1186/s13567-019-0651-1)
Supplement: Supplementary file 2 — Additional file 2. In-silico experiments to test the modelling framework in fully known populations. This file contains the protocol and results of the in silico experiments conducted to test our modelling framework. We constructed fully known populations in which we varied the values of four key parameters in order to investigate their influence on the estimation of vertical and vector-borne transmission: the probability of vertical transmission in the population, the size of the dataset, the level of exposure to vector-borne transmission, and the spatial heterogeneity of that exposure in the study area. We thus identified a set of conditions providing reliable estimates of vertical transmission. [file 13567_2019_651_MOESM2_ESM.docx]

**Additional file 2:** *In-silico* experiments to test the modelling framework in fully known populations.

**METHODS**

We tested our framework with a series of toy examples. We constructed fully known populations in which we varied the values of four key parameters in order to investigate their influence on the estimation of vertical and vector-borne transmission: the probability of vertical transmission in the population, the size of the dataset, the level of exposure to vector-borne transmission, and the spatial heterogeneity of that exposure in the study area. We did not investigate the temporal heterogeneity of exposure to vector-borne transmission as we did not detect any significant difference in the temporal distribution of RT-PCR+ results of calves <3months in the area where most of the 2016 BTV-8 circulation occurred.

We generated a synthetic population of finite size N=15 000 with the same distributions of age classes and sampling dates as that of the population of sampled cattle. We applied the same known monthly forces of vector-borne infection to calves and dams: from June 2016, or their birthdate if later, to their sampling date, for calves; from June 2016 to parturition date for dams.

We compared three exposure patterns to vector-borne infection. In all of them, we assumed that there would be a peak in the probability of infection with most transmission occurring in September and October, as suggested by the distribution of RT-PCR results. We varied the size of the peak, *i.e.* the level of exposure, in each of these scenarios, called H, M, and L, for high, medium and low-level of exposure respectively (Figure AF.2.A-C.). We fixed the size of the peak in the two extreme scenarios based on a previous study in which we used similar catalytic models to study the evolution of the probability of seroconversion in French cattle from the emergence to the re-emergence [33]: the scenario of high exposure resulted in a peak higher than the highest reconstructed in that study, while the low-level scenario resulted in the smallest distinguishable peak.

We first considered the same exposure pattern to vector-borne infection for all cattle and explored three “homogeneous exposure scenarios”: 100% exposure to H, M or L, respectively. To simulate spatial heterogeneity of virus circulation, we also explored five “heterogeneous exposure scenarios”: 33% exposure to each pattern, 70% exposure to a pattern and 15% to the two others (three combinations), 50% exposure to H and M; and we randomly selected cattle exposed to each pattern given these proportions.

Calves born before June 2016 were additionally exposed to vertical transmission from infected gestating heifers, and we applied a probability of 50% vertical transmission. We varied the probability of vertical transmission (γ=25%, 75%), and decreased the size of the synthetic population (N=10 000; 5 000; 2 500) in the homogeneous exposure scenarios.

We then computed animal-level probability of testing RT-PCR positive in each scenario and simulated sets of RT-PCR results by successive binomial draws. We used these simulated datasets to estimate vertical transmission and monthly probabilities of vector-borne infection that we compared to the input ones.

In the heterogeneous exposure scenarios, we compared two models: *model one-area*, that did not account for spatial heterogeneity in exposure and allowed reconstructing a unique set of monthly probabilities of vector-borne infection in the whole area and a unique probability of vertical transmission; and *model sub-areas*, in which the whole area was sub-divided into areas grouping together animals with similar exposure patterns allowing the reconstruction of distinct probability sets of vector-borne infection for each sub-area, but the probability of vertical transmission for all sub-areas was unique.

In these scenarios where cattle from the various sub-areas had been exposed differently to vector bites, we needed a measure to approximate the global level of exposure to vector-borne infection in the whole study area. We thus used the RT-PCR proportion of positive results (RT-PCR+ %) in cattle only exposed to vector bites, calculated over the whole period from June to December 2016, as a proxy of the cumulative exposure to infectious bites (Table AF2.1).

**RESULTS**

### *Impact of the level of vector-borne virus circulation, probability of vertical transmission and dataset size in the homogeneous exposure scenarios*

In the high and medium homogeneous exposure scenarios to vector-borne infection, we correctly estimated the average probability of vertical transmission (γ) in the synthetic population (Figure AF.1.A-B). The mean estimated γ values matched the input value for the three probabilities tested (γ=25%, 50%, 75%, Figure AF.1.A) and for all dataset sizes (N=15 000, 10 000, 5 000, 2 500, Figure AF.1.B). We obtained similar widths of credible intervals (CI95%) whatever the input probability of vertical transmission and for a given level of exposure (Figure AF.1.A). We observed an impact of population size and of the level of exposure to infectious bites on confidence levels, with an increase in the width of credible intervals for decreasing sizes (Figure AF.1.B) and for decreasing exposure levels (Figure AF.1.A-B).

In the low exposure scenario, credible intervals covered >70% of the parameter space, with mean values diverging from the input ones for all γ probabilities (Figure AF.1.A) and sample sizes tested (Figure AF.1.B).

Hence, both the level of exposure to infectious bites and the size of the dataset impacted parameter estimation with an interaction effect, so that the increase in the width of credible intervals with decreasing dataset sizes was greater in the medium *vs* high exposure scenario (Figure AF.1.B). In the latter, the credible interval covered <25% of the parameter space even with the smallest dataset (Figure AF.1.B), whereas in the medium exposure scenario, it reached >50% of the parameter space for N=2 500, though the mean estimate of vertical transmission remained similar to the input one.

### *Impact of the spatial heterogeneity of vector-borne virus circulation*

In the eight scenarios where cattle from the various sub-areas had been exposed differently to vector bites, we first ignored heterogeneity in exposure within the study area (*model one-area*). Inference results were sorted by global levels of exposure, using the RT-PCR proportion of positive results in cattle only exposed to vector bites, calculated over the whole period from June to December, as a proxy of the cumulative exposure to infectious bites (Figure AF.1.C). We observed an increase in credible intervals of estimated γ values for the scenarios with lower global levels exposure, *i.e.* when RT-PCR+% dropped under 10%. Credible intervals kept increasing with lower proportions (Figure AF.1.C).

We then accounted for area-specific patterns of exposure to vector-borne infection with the *model sub-areas* (Figure AF.1.D, Figure AF.2.D-H)*.* This approach allowed highlighting local contrasts by reconstructing area-specific monthly probabilities of vector-borne infection (Figure AF.2.D-H). Regarding vertical transmission, we estimated identical average probabilities with both models (Figure AF.1.D). Therefore, inference was similar when considering cattle from a unique area with heterogeneous exposure or from sub-areas in which exposure was homogeneous.

**Table AF2.1:** Proportion of RT-PCR positive results in each scenario of the *in-silico* experiments.

| **Proportion of RT‑PCR+ results (%)** | | **Homogeneous exposure** | | | **Heterogeneous exposure** | | | | |
| --- | --- | --- | --- | --- | --- | --- | --- | --- | --- |
|  |  | H | M | L | 33% HML | 70%H | 70%M | 70%L | 50% H/M |
| In those only exposed to vectors (N=5 430) | | 25.8 | 8.36 | 0.91 | 12.01 | 19.46 | 9.97 | 5.71 | 17.19 |
| In all cattle  (N=15 000) | γ=0% | 15.58 | 5.26 | 0.57 | x | x | x | x | x |
|  | γ=25% | 18.95 | 7.26 | 0.68 | x | x | x | x | x |
|  | γ=50% | 22.12 | 7.29 | 0.80 | 9.11 | 15.38 | 7.74 | 4.37 | 13.17 |
|  | γ=75% | 25.46 | 8.47 | 0.95 | x | x | x | x | x |

The proportions of RT-PCR positive results were calculated over the whole study period from June to December 2016, in a synthetic population (N = 15 000) under eight scenarios of exposure to vector-borne transmission, and with four probabilities of vertical transmission. The results were averaged over ten toy datasets generated for each scenario. γ: probability of vertical transmission. H, high; M, medium; L, low. 33% HML: 33% exposure to each pattern; 70%H - 70%M - 70%L: 70% exposure to a pattern and 15% to the two others; 50% H/M: 50/50% exposure to H and M; x: scenario not investigated in the *in-silico* experiments.

**Figure AF.1:** Results of the *in-silico* experiments**.**


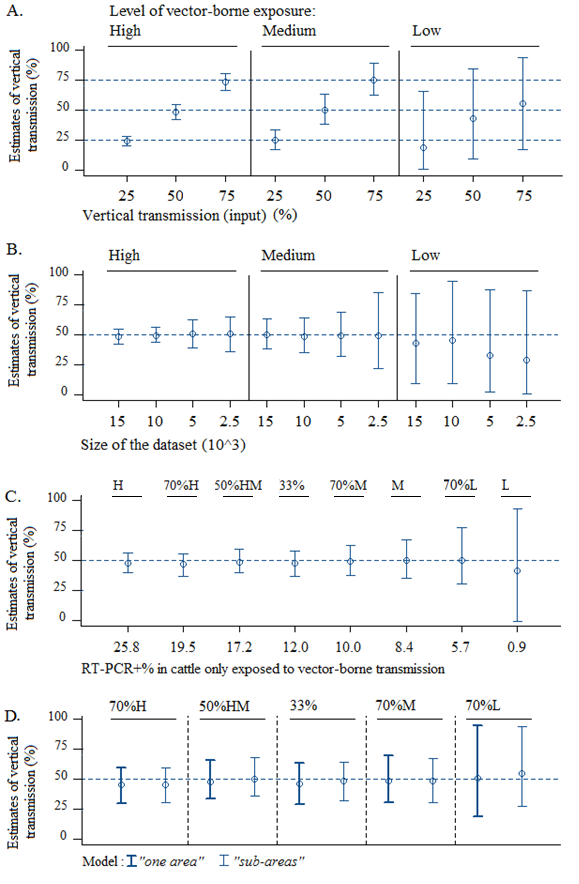


Impact on the estimation of vertical transmission of four parameters: A. the level of exposure to vector-borne transmission and the probability of vertical transmission in the population; B. the level of exposure to vector-borne transmission and the size of the dataset; C. the level of exposure to vector-borne transmission; D. the spatial heterogeneity of that exposure in the study area. The scenarios of exposure to vector-borne transmission are specified on top of each graph. Unless specified otherwise, a probability of vertical transmission of 50% was applied to a synthetic population of size N=15 000 and predicted vertical transmission probabilities (CI95%) were reconstructed from that population with model *one-area*. Homogeneous exposure patterns of vector-borne infection: 100% exposure to high (H), medium (M) and low-level (L) of vector-borne circulation. Heterogeneous exposure patterns of vector-borne infection: 33% exposure to each pattern (33%), 70% exposure to a pattern and 15% to the two others (70%H, 70%M, 70%L), 50% exposure to H and M (50%HM).

**Figure AF.2:** Monthly probabilities of vector-borne infection reconstructed in the scenarios of heterogeneous exposure.


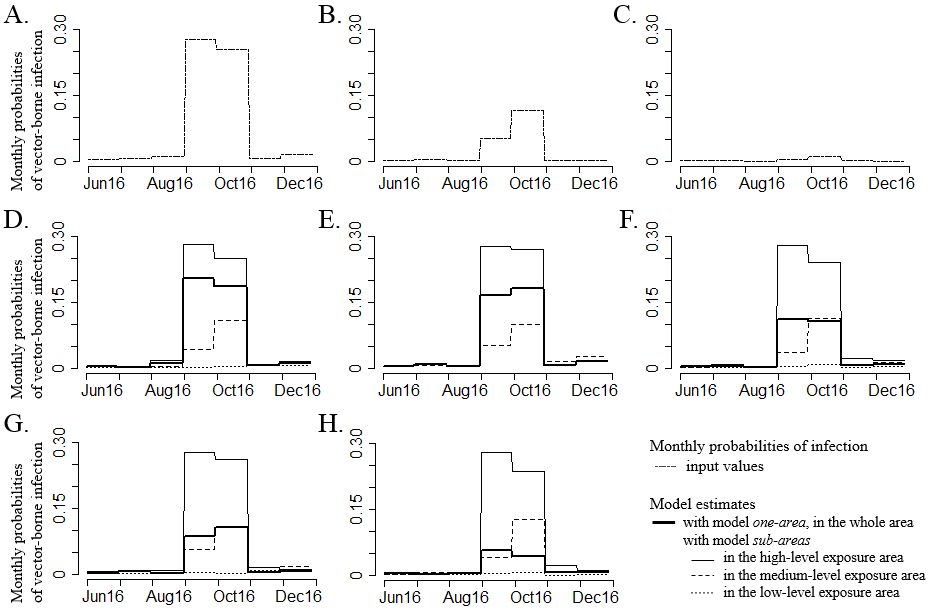


The monthly probabilities of vector-borne infection were reconstructed in the scenarios of heterogeneous exposure with *model one-area vs model sub-areas*. A-C. Input values for each homogeneous exposure scenario: high (A), medium (B) and low (C). D-H. Inference results for the five heterogeneous scenarios of exposure to vector-borne infection: 70%H (D), 50%HM (E), 33% (F), 70%M (G), 70%L (H). With model *one-area*, there is only one line representing the probability of vector-borne infection reconstructed in the whole area, while with model *sub-areas*, there are several lines: one for each sub-area. Heterogeneous scenarios: 33% exposure to each pattern (33%), 70% exposure to a pattern and 15% to the two others (70%H, 70%M, 70%L), 50% exposure to H and M (50%HM).
