# Supplementary material for: Quantifying bluetongue vertical transmission in French cattle from surveillance data
Source: Vet Res. 2019 May 14;50:34. doi: 10.1186/s13567-019-0651-1 (PMC6518818; doi:10.1186/s13567-019-0651-1)
Supplement: Supplementary file 3 — Additional file 3. Convergence of the chains obtained for the seven estimated parameters of model one-area. In this file, we checked the convergence of the chains obtained for the seven parameters. We first provided visual indicators of the convergence of the chains obtained for the seven parameters of model one-area in the whole study area. Then, we provided two convergence statistics for these chains: Rhat, that is the potential scale reduction factor and Neff, that is the effective number of samples. [file 13567_2019_651_MOESM3_ESM.docx]

**Additional file 3 Convergence of the chains obtained for the seven estimated parameters of model *one-area*.**

**Figure AF3.1.** Visual indicators of convergence: MCMC chains obtained for the seven parameters of model *one-area* in the whole study area: one *γ* (A) and 7 *logλ_j_* (B-H), one per month *j* from June 2016 to December 2016, i.e. parameter values plotted in the 1500 iterations kept after the burning period.


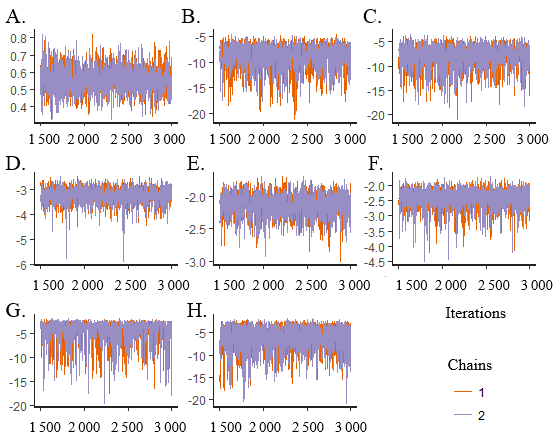


**Table AF3.1.** Convergence statistics for the MCMC chains obtained for the seven parameters of model *one-area* in the whole study area: one *γ* and 7 *logλ_j_*, one per month *j* from June 2016 to December 2016. Rhat is the potential scale reduction factor; Neff is the effective number of samples.

| **Parameter** | ***γ*** | ***logλ_1_*** | ***logλ_2_*** | ***logλ_3_*** | ***logλ_4_*** | ***logλ_5_*** | ***logλ_6_*** | ***logλ_7_*** |
| --- | --- | --- | --- | --- | --- | --- | --- | --- |
| Neff | 1 231 | 1 052 | 1 115 | 1 434 | 1 302 | 859 | 688 | 1 317 |
| Rhat | 1.001 | 1.000 | 1.002 | 1.000 | 1.001 | 1.000 | 1.000 | 1.003 |
